# A Fatal Bloodstream Infection by *Staphylococcus pettenkoferi* in an Intensive Care Unit Patient

**DOI:** 10.1155/2011/612732

**Published:** 2011-10-20

**Authors:** Caterina Mammina, Celestino Bonura, Maria Stella Verde, Teresa Fasciana, Daniela Maria Palma

**Affiliations:** ^1^Department of Sciences for Health Promotion “G. D'Alessandro”, Section of Hygiene, University of Palermo, Via del Vespro 133, Palermo 90127, Italy; ^2^Department of Sciences for Health Promotion “G. D'Alessandro”, Section of Microbiology, University of Palermo, Via del Vespro 133, Palermo 90127, Italy; ^3^Laboratory of Microbiology, General Hospital ARNAS “Civico, Di Cristina & Benfratelli”, Piazza Nicola Leotta, Palermo 90127, Italy; ^4^II Intensive Care Unit, General Hospital ARNAS “Civico, Di Cristina & Benfratelli”, Piazza Nicola Leotta, Palermo 90127, Italy

## Abstract

Coagulase negative staphylococci are increasingly recognized as leading pathogens in bacteremia, with incidence peaking in intensive care units. Interpretation of blood cultures that are positive for CoNS is often doubtful. We describe a fatal case of bacteremia by a newly recognized species of CoNS, *Staphylococcus pettenkoferi*, in an ICU patient.

## 1. Introduction

Coagulase negative staphylococci (CoNS) are increasingly recognized as leading pathogens in bacteremia, especially in patients with indwelling or implanted devices, such as intravenous catheters, prosthetic heart valves, and joint prostheses, or in those immunocompromised [1, 2]. Their capacity to adhere to inert surfaces along with the wider use of vascular devices accounts for generally high incidence rates varying according to the type and size of the institution and wards and peaking in intensive care units (ICUs) [3].

*Staphylococcus pettenkoferi* (*S. pettenkoferi*) is a newly recognized member of the CoNS. Its identification was first reported in 2002 from a blood culture of a young patient with extrapulmonary tuberculosis and from a wound swab of an elderly patient with multiple comorbidities [4]. More recently, three more isolates of *S. pettenkoferi* from blood cultures of different patients in Belgium and Germany have been reported [5]. Two strains have been described in 2007 in a French diabetic patient with osteomyelitis and in a case of bloodstream infection in Korea in 2009, respectively [6, 7]. In 2010, two further isolates have been identified from a patient hospitalized in Porto Alegre, Brazil [8]. 

In this study, we describe a fatal case of bloodstream infection where *S. pettenkoferi *was identified as the only pathogenic organism from blood cultures. 

## 2. Case Presentation

The 49-year-old male patient was admitted on April 8, 2011 to the II ICU of the General Hospital ARNAS “Civico, Di Cristina & Benfratelli”, Palermo, Italy, for the surgical treatment of a posttraumatic hydrocephalus. The patient had been transferred from a long-term care rehabilitation center (LTCRC), where he was staying over a six-month period after a head injury. He presented at our ICU in coma, in spontaneous breathing trough a tracheostomy and with enteral feeding trough a percutaneous endoscopic gastrostomy (PEG). On day 8, he underwent to the placement of a ventriculo-peritoneal device (VPD). On day 18, this VPD was replaced by an external ventricular drainage due to the occlusion of the internal device. The management of the patient was complicated by an infection of the external drainage caused by a carbapenem-resistant strain of *Klebsiella pneumoniae*, which was treated with intravenous and intrathecal colistin sulphomethate sodium. A decrease in the cerebrospinal fluid (CSF) white blood cell count was obtained and, six days after starting colistin, bacterial cultures tested negative and the patient's clinical situation improved. Ten days later, the patient's condition deteriorated again and he developed a septic shock. Mechanical ventilation was begun, and norepinephrine was administered. Blood, urine, bronchoalveolar lavage, and CSF samples for culture were obtained. Empirical antibiotic treatment was started with daptomycin (8 mg/Kg) plus piperacillin-tazobactam (4.5 g × 4) because a bloodstream infection was suspected. Two blood cultures obtained within 48 hours from each other grew a methicillin resistant coagulase negative *Staphylococcus* spp. isolate. Consequently, the antibiotic treatment with daptomycin was confirmed and piperacillin-tazobactam discontinued. Nonetheless, the patient developed cardiac arrest and efforts to resuscitate him were unsuccessful. 

The two isolates were initially characterized by means of the Vitek2 identification system (BioMérieux, Marcy l'Etoile, France) as *S. capitis*. However, biochemical analysis with the API Staph system (BioMérieux, Marcy l'Etoile, France) attributed the isolates with a doubtful profile as *Staphylococcus capitis, *probability 71.5%, or other species with a probability lower than 25%. Because of these inconsistent biochemical findings, we proceeded to the sequencing of the 16S rRNA gene. The 16S rRNA genes from both isolates were amplified by PCR (Perkin-Elmer Gene-Amp PCR System 2720) using the universal primers 27F 5′-AGAGTTTGATCMTGGCTCAG-3′ and 1492R 5′-TACGGYTACCTTGTTACGACTT-3′ [8]. PCR products were purified and sequenced using the BigDye Terminator v1.1 Cycle Sequencing kit (Applied Biosystems Warrington, UK) and the ABI Prism 310 Genetic Analyzer (Applied Biosystems, Foster City, Calif, US). 16S rRNA was sequenced twice to ensure sequence data accuracy. Sequence data were compared with NCBI GenBank entries by using the BLAST algorithm (http://www.ncbi.nlm.nih.gov/BLAST/) and showed 100% similarity with sequences from *S. pettenkoferi* strain B3117 (GenBank accession number AF322022) and *S. pettenkoferi* strain A6664 (GenBank accession number DQ538520) previously determined by Trülzsch et al. [5]. 

*In vitro* susceptibility tests using the VITEK 2 system showed that the two methicillin resistant isolates were resistant to beta-lactams, erythromycin, clindamycin, fosfomycin, gentamicin, and tobramycin and susceptible to ciprofloxacin, daptomycin, moxifloxacin, netilmicin, sulfamethoxazole-trimethoprim, rifampicin, fusidic acid, teicoplanin, tygecycline, vancomycin, and mupirocin. 

## 3. Discussion

The present paper provides further evidence to the pathogenic role of a recently recognized species of CoNS, *S. pettenkoferi*. According to the general rule for CoNS and many other opportunistic organisms, a possible contamination from the patient saprophytic flora or the environment has to be reasonably ruled out before attributing an etiological role [9]. Also of interest, *S. pettenkoferi* has been previously detected in the indoor environment by direct analysis of the 16S rRNA gene from dust samples [10]. However, criteria that have been developed to help distinguishing between contamination and true bacteremia are not so straightforward and all have a low positive predictive value [11]. In particular, two or more positive culture results have a high positive predictive value only when blood samples are not obtained through a catheter [12]. Moreover, associated mortality has been proved to be similar among patients with one positive blood culture and those with two or more positive blood cultures [9]. In our case, the worsening clinical conditions of the patient had no alternative explanation and the isolates from the two culture bottles shared an identical 16S rRNA gene sequence. Consequently, in the light also of the peculiar CoNS species involved, the etiological role of *S. pettenkoferi* could be plausibly considered. 

As it has been previously supposed, the exceedingly infrequent identification of such a species of CoNS is likely to be attributed to the inability of the clinical microbiology laboratories to accurately identify this organism by using automated or commercial identification systems [7]. The consequent misidentification does not allow *S. pettenkoferi* to be correctly categorized as the likely causative agent of an infectious event and a helpful support to clinical decisions to be provided. On the other hand, it can also hinder the recognition of a transmission chain of infections in a healthcare setting. Molecular methods give the opportunity to timely and effectively overcome these drawbacks. 
